# Tackling Multiple-Drug-Resistant Bacteria With Conventional and Complex Phytochemicals

**DOI:** 10.3389/fcimb.2022.883839

**Published:** 2022-06-30

**Authors:** Thangaiyan Suganya, Issac Abraham Sybiya Vasantha Packiavathy, G. Smilin Bell Aseervatham, Areanna Carmona, Vijayaragavan Rashmi, Subramanian Mariappan, Navaneethan Renuga Devi, Devanesan Arul Ananth

**Affiliations:** ^1^ Department of Microbiology, Karpagam Academy of Higher Education, Coimbatore, India; ^2^ Department of Biotechnology, Karpagam Academy of Higher Education, Coimbatore, India; ^3^ Post Graduate Research Department of Biotechnology and Bioinformatics, Holy Cross College (Autonomous), Tiruchirappalli, India; ^4^ Francis Graduate School of Biomedical Sciences, Texas Tech University Health Science Center of El Paso, Texas, TX, United States; ^5^ National Repository for Microalgae and Cyanobacteria (NRMC)- Marine, National Facility for Marine Cyanobacteria, (Sponsored by Department of Biotechnology (DBT), Government of India), Bharathidasan University, Tiruchirappalli, India; ^6^ Post Graduate and Research Department of Zoology, Yadava College, Madurai, India; ^7^ Department of Zoology, GTN Arts College, Dindigul, India

**Keywords:** Multi Drug Resistant (MDR), phytochemicals, bacteria, pathogenesis, antibiotics

## Abstract

Emerging antibiotic resistance in bacteria endorses the failure of existing drugs with chronic illness, complicated treatment, and ever-increasing expenditures. Bacteria acquire the nature to adapt to starving conditions, abiotic stress, antibiotics, and our immune defense mechanism due to its swift evolution. The intense and inappropriate use of antibiotics has led to the development of multidrug-resistant (MDR) strains of bacteria. Phytochemicals can be used as an alternative for complementing antibiotics due to their variation in metabolic, genetic, and physiological fronts as well as the rapid evolution of resistant microbes and lack of tactile management. Several phytochemicals from diverse groups, including alkaloids, phenols, coumarins, and terpenes, have effectively proved their inhibitory potential against MDR pathogens through their counter-action towards bacterial membrane proteins, efflux pumps, biofilms, and bacterial cell-to-cell communications, which are important factors in promoting the emergence of drug resistance. Plant extracts consist of a complex assortment of phytochemical elements, against which the development of bacterial resistance is quite deliberate. This review emphasizes the antibiotic resistance mechanisms of bacteria, the reversal mechanism of antibiotic resistance by phytochemicals, the bioactive potential of phytochemicals against MDR, and the scientific evidence on molecular, biochemical, and clinical aspects to treat bacterial pathogenesis in humans. Moreover, clinical efficacy, trial, safety, toxicity, and affordability investigations, current status and developments, related demands, and future prospects are also highlighted.

## Introduction

### Origin of Antibiotic Resistance

Bacterial penicillinase was discovered by two members of the penicillin discovery team many years before the use of penicillin as a healing agent as discovered by Alexander Fleming in 1928. Resistant strains that deactivated the drug emerged as a result of the extensive use of antibiotics. Consequently, research to chemically acclimatize penicillin to stop cleavage by penicillinases (-lactamases) began (D’Costa et al., 2006). Following penicillin, streptomycin came to practice in 1944 for the treatment of tuberculosis (TB) (TB Alliance, 2019). During the course of clinical practice with streptomycin, resistant strains of *Mycobacterium tuberculosis* developed. Even though innovative findings of streptomycin and isoniazid were used to fight TB, there was still rapid progress in resistance. The administration of anti-TB drugs in cocktail form has developed as an important therapeutic routine with notable recovery; however, multidrug resistance remains constant to TB treatment throughout the world for a variety of reasons. In the last two decades, *M. tuberculosis* strains have become extremely drug-resistant (XDR) to front-line antibiotics, including ethionamide, para-amino salicylic acid, cycloserine, ofloxacin, amikacin, and ciprofloxacin. Later, they may become totally drug-resistant strains (Velayati et al., 2009). The evolution of multidrug resistance in *M. tuberculosis* through horizontal gene transfer (HGT) is not evidenced by any authenticated research. Hence, it is predicted that antibiotic resistance in *M. tuberculosis* might be attributed to spontaneous mutation.

Similarly, the most common Gram-negative pathogens, like *Escherichia coli*, *Salmonella enterica*, and *Klebsiella pneumoniae*, cause many diseases in humans. Since the past half-century, antibiotic resistance development was observed towards these diseases due to antibiotic misuse and overuse. Particularly, the lactam group of antibiotics and their associated inactivating lactamase enzymes are more prevalent; nearly 1,000 resistant lactamase groups have been reported (Livermore et al., 2006). The development and transmission of resistance to lactam antibiotics among enteric groups of bacteria in the community as well as in hospital infections is majorly increased by HGT. Another major nosocomial pathogen, *Pseudomonas aeruginosa*, originated from a burn wound infection in which the antibiotic resistance mechanisms progressed accidentally due to treatment with new antibiotic derivatives over the existing lactam and aminoglycosides. *P. aeruginosa* is extremely difficult for patients infected with cystic fibrosis since the pathogen is extremely persistent and has the ability to bypass the human defense mechanism. Prolonged antibiotic regime among cystic fibrosis patients is closely linked with resistance development.

*Acinetobacter baumannii*, a Gram-negative nosocomial pathogen, causes severe mortality and morbidity due to its R genes and pathogenicity factors (Peleg et al., 2008). Acinetobacter obtained pathogenic determinants due to their dynamic existence and biodegradation abilities in harsh environments; moreover, several strains in nature are competent for DNA uptake and have a high chance of spontaneous transformation. Recent molecular research reported that *A. baumannii* has rapid evolution, with a minimum of 28 genomic islands encrypting antibiotic resistance determinants; additionally, 50% of these inserts also translate virulence in the form of type IV secretion systems (Gomez and Neyfakh, 2006).

*Staphylococcus aureus*, a Gram-positive superbug, is highly associated with the human population as nasal commensal in 30% of the population, and its occurrence is linked with common skin infections. Unlike *M. tuberculosis*, it does not have a strong historical status, but *S. aureus* has developed as a major multidrug-resistant nosocomial infection (Enright et al., 2002). After the discovery of penicillin, *S. aureus* infections became manageable, but the strain developed resistance over the course of time. The innovative discovery of methicillin in 1959 was assumed to be an effective antibiotic against penicillinases, but within 3 years, the methicillin-resistant *S. aureus* (MRSA) developed. At present, MRSA has started to transfer, with higher virulence and transmission features, outside the hospital and stands as a major community-acquired pathogen (DeLeo and Chambers, 2009).

Due to the frequent use of antibiotics, the majority of epidemic bacterial pathogens related to human disease developed into multidrug-resistant (MDR) strains. “Superbugs” is the term given to describe microbes with higher morbidity and mortality due to numerous mutations. These “superbugs” result in increased resistance to antibiotics exactly prescribed for their treatment. Thus, the healing choices for these microbes become less with prolonged hospitalization. Sometimes the “superbugs” attained enhanced virulence and a higher level of transmission. As a result, antibiotic resistance was considered a potential virulence factor (Davies and Davies, 2010).

### Carriers of Antibiotic Resistance

Understanding of several carriers of antibiotic resistance is an important fact needed to face the global problem. The essential features which are potential carriers of antibiotic resistance include sanitation settings, infection control standards, water quality, standard of drug, diagnostics and treatments, and migration quarantine. Apart from mutations, in diverse genes of the bacterial chromosome, the direct transfer of genetic material between organisms plays an important role in the circulation of antibiotic resistance. The transfer of plasmid among bacteria is one of the vital features which may transfer genes of antibiotic resistance to the host cell (Holmes et al., 2016). Antibiotics may influence this process by inducing the transmission of resistance elements; furthermore, they employ selective pressure to the development of resistance (Munita and Arias, 2016).

Sometimes the environment can offer a path for resistant bacteria to form colonies or infect host organisms (Mazel, 2006). This is referred to as “transmission event”, while variations in their DNA sequence as well as genetic transmission among bacterial species are considered as “evolution events”. In the case of a resistant pathogen that is already common among humans, the significance of a single transmission to one more person is more restricted than for an evolutionary event, resulting in the advent of a new, potential resistance genotype in pathogens with hypothetically global significances. Even though few pathogens, like *Vibrio* spp., survive in the environment, it is a comparatively unfriendly environment than a human or domestic animal host. Hence, development in the environment is quite limited for those kinds of pathogens. It is possible that minor growth changes between resistant and non-resistant strains, triggered by sub-minimum inhibitory concentration (MICs) of antibiotics, are a minor factor for the opportunity that environmental exposure becomes adequately enough for the colonization or infection of a host. The rest of the living and non-living features like temperature, oxygen pressure, nutrients, predation, and competition with other species, all discrete to the antibiotic resistance habit of the bacteria, are possible to be more significant for environmental transmission chances for both resistant and non-resistant bacterial strains (Larsson and Flach, 2021).

Basically, additional genetic elements present in bacteria have the capacity of up-taking resistance genes and helping their transmission; based on the genus of the pathogen, the nature of genetic factors differs. Plasmid-mediated resistance transmission is the most common mode of HGT (Norman et al., 2009). Unexpectedly, bacteriophages taking up antibiotic resistance genes have been reported in the environment or from resistant bacteria found in hospitals; there is still no inquiry about the connection of phages with the insertional mechanisms essential for the development of mobile resistance factors or with the functions of chromosomally linked genes. Usually, they are termed as “fingerprints”, flanking genes encrypting resistance or virulence on various vectors. These actions are found to be quite common in *S. aureus*. Among bacterial genera like *Streptococci*, *Meningococci*, and other related genera, the exchange of both virulence and pathogenicity genes is unlimited. The main mode of DNA transfer is found to be transformation (Springman et al., 2009). *Acinetobacter* spp. is competent in nature to uptake DNA directly from the environment with frequent HGT (Barbe, 2004) because pathogenic bacterial strains transfer large genomic islands (Perez et al., 2007). Throughout the history of bacterial evolution, HGT has occurred; two independent sets of actions should be taken into account, which is mainly distinguished by their time span and the strength of selection pressure. Bacterial evolution over billions of years cannot be related to the mode of antibiotic resistance development and transfer over the last century. The selection pressure of intense antibiotic treatment and clearance is even higher; the selection is majorly necessary for existence in hostile environments rather than for features offering resistance in gradually developing groups of populations.

### Genetic Insights of Antibiotic Resistance

Bacterial resistance towards antibiotics might be native, a unique feature of specific bacterium which is based on its biological phenomena, whereas acquired resistance is obtained through (i) the attainment of exogenous genes by plasmids through conjugation, transposons (conjugation), integrons, and bacteriophages through transduction, (ii) gene mutation, and (iii) a blend of the above-mentioned processes (Mims et al., 2004). Generally, chromosomal mutations are occasional and control resistance to structurally similar compounds (Rice et al., 2003). These kinds of spontaneous mutations take place as mistakes during replication or a damaged DNA that escaped from the repair system. The antibiotic resistance of *E. coli* against quinolones developed due to alterations in a minimum of seven and three amino acids in the *gyrA* gene or *parC* gene, respectively (Džidić et al., 2008). In contrast, only a single-point mutation in the *rpoB* gene is related to a wide-ranging resistance to rifampin (Rice et al., 2003). Through mutation, antibiotic uptake or efflux system can be altered (Hooper, 2001). Adaptive mutations take place only during the nonlethal selection of microorganisms. In this mutation, the new gene holder gets deleted at a specific recombination site (*attI* site) and at a promoter that starts gene transcription. The majority of class I integrons in the 3′ conserved segment has a supplementary gene *suII* accountable for resistance to sulphonamides (Hooper, 2001; Daikos et al., 2007).

Out of 21 reported anti-microbial resistance (AMR) genes, the vital genes accountable for MDR *Salmonella* and *E. coli* are *AmpC*, *bla-TEM-1*, *bla-CTXM-15*, *VIM-1*, *NDM-1*, *floR*, and *tetG* and the recently found *mcr-1* gene with resistance to colistin. Diverse modes of resistance and new transmission vectors and genes are reported consistently. Bacteria carry two mechanisms for resistance, known as intrinsic resistance and acquired resistance (Lynch et al., 2013). The capacity of a bacterium to overcome the attack of a particular antibiotic by innate structural or functional phenomena is called intrinsic resistance. *Pseudomonas* is an outstanding example of an intrinsic resistance mechanism because of the absence of a vulnerable target site for a specific antibiotic. Triclosan is a versatile antibiotic, particularly against Gram-positive bacteria and several Gram-negative bacteria, but is unable to control the growth of *Pseudomonas.* Besides this, they are highly resistant to aminoglycosides, quinolones, and β-lactams.

**Figure 1:**
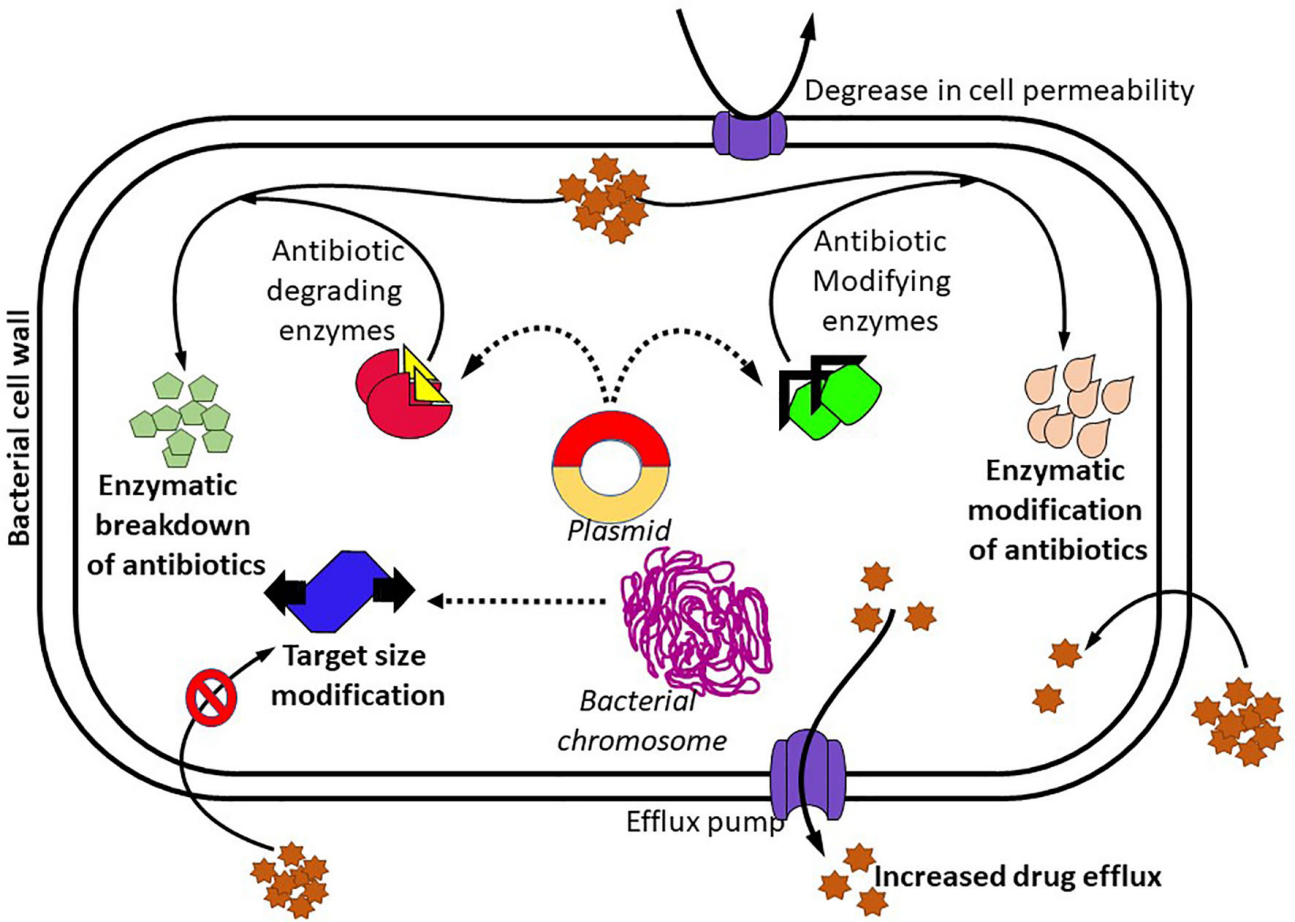
Molecular mechanisms of bacteria resisting antibiotics.

Moreover, various other processes have also been reported to be involved in microbial resistance against an antibiotic, including the upregulation of efflux pumps, structural modification of porins, enzyme synthesis, and cell-to-cell communication (Porras et al., 2020), and this is represented in **Figure 1**. Membrane proteins that have the ability to transfer antibiotics from the cell, thereby sustaining their low intracellular concentrations, are known as efflux pumps. When the permeability of the outer membrane (OM) gets lowered, the antibiotic uptake also gets reduced (Džidić et al., 2008). Assessment of efflux pumps is one of the most crucial factors in the analysis of antibiotic resistance. In single-component efflux systems, substrates are passed through the cytoplasmic membrane, but in Gram-negative bacteria, multicomponent pumps and a periplasmic membrane synthesis protein component transfer the substrates through the cell envelope (Alekshun and Levy, 2007; Džidić et al., 2008). Efflux pumps can be unique to each type of antibiotic. The majority of them are multidrug transporters that have the ability to pump various antibiotics like macrolides, tetracyclines, fluoroquinolones; thereby, it remarkably offers to MDR (Džidić et al., 2008). Frequently, bacteria resistant to tetracyclines secrete higher levels of membrane proteins which are used as efflux pumps for antimicrobial drugs. To remove toxic compounds from the cytoplasm and periplasm, *P. aeruginosa* utilizes more than four potential MDR efflux pumps (Strateva and Yordanov, 2009). MDR efflux pumps like MexV-MexW-OprM are responsible for resistance to antibiotics such as fluoroquinolones, tetracyclines, chloramphenicol, erythromycin, and acriflavine (Strateva and Yordanov, 2009). The higher-level expression of MexAB-OprM efflux pumps leads to increased inhibitory concentration against antibiotics like penicillins, cephalosporins, chloramphenicol, fluoroquinolones, macrolides, novobiocin, sulfonamides, tetracycline and trimethoprim, dyes (SYBR safe, Gelgreen), and detergents (SDS, Triton X-100) (Thomson and Bonomo, 2005). In Gram-negative bacteria, the β-lactam antibiotics can pass through a membrane protein occupied with a water molecule termed porin. When *P. aeruginosa*-specific OprD2 porin is absent, it results in resistance to imipenem, whereas resistance to meropenem takes place due to variations in the MexAB-OprM efflux system (Bradford, 2001; Džidić et al., 2008). Bacterial genera like *Enterococcus aerogenes*, *Klebsiella* spp., *Proteus mirabilis*, *Serratia marcescens*, *Morganella morganii*, *H. influenzae*, and *Helicobacter pylori* are reported to have homologs of *Mex* and *Acr* efflux systems (Piddock, 2006). The chief elimination system for macrolides, which is encrypted by the *mef* gene, is predominant in Gram-positive bacteria that is used for the removal of fluoroquinolones and aminoglycosides from the bacterial cell. *E. coli* and *K. pneumoniae* are comprised of an elimination system of tetracyclines and chloramphenicol, which is encoded by *ramA* gene. The same phenomena provide antibiotic resistance to norfloxacin (Grundmann et al, 2006).

The OM of Gram-negative bacteria encompasses an internal layer that contains phospholipids and an external layer that has the lipid A molecule. Hence, the nature of OM arrangement lessens drug uptake to a cell and passes *via* the OM. Antibiotics are transferred to a cell by the following mechanisms: (i) diffusion *via* porins, (ii) diffusion across the bilayer, and (iii) self-influenced uptake. The mode of transport is mainly based on the chemical composition of an antibiotic (Džidić et al., 2008). The reduced OM permeability of *P. aeruginosa* provides acquired resistance to multiple antibiotic groups. Little hydrophilic molecules, like β-lactams and quinolones, can pass through the OM only *via* porins. Acquired resistance is a distinctive feature of maximum resistance to almost all aminoglycosides, particularly to tobramycin, netilmicin, and gentamicin (Ferguson et al., 2007). Bacterial quorum sensing (QS), also called cell-to-cell communication, helps chemical signals, called autoinducers, activate to regulate pathogenic behaviors and assist bacteria to escape from antibiotics and host immune response. The three types of QS signals in bacteria are acyl-homoserine lactone, auto-inducing peptide, and autoinducer-2. QS signaling activation and subsequent biofilm formation lead to the antimicrobial resistance of the pathogens, thus increasing the therapy difficulty of bacterial diseases (Jiang et al., 2019).

### Consequences of Antibiotic Resistance

Antibiotic-resistant bacteria are also termed as superbugs. The anxiety created by these organisms is not only relevant for the laboratory but has also emerged as a global risk responsible for the high death rate and lethal infections (Lipp et al., 2002). According to predicted statistical models, bacterial AMR caused an estimated 495 million deaths in 2019, with 127 million (95%) deaths attributed to bacterial AMR (Antimicrobial Resistance Collaborators, 2022). World Health Organization (WHO) has cautioned that a post-antibiotic period will be affected with infections often, and even minor wounds may lead to death if antibiotic resistance is not addressed properly. Multidrug-resistant bacteria cause more deaths worldwide. Several countries are fronting the problem of nosocomial infections through *S. aureus* as waves of clonal distribution. All over the world, MRSA strains are reported to be quickly spreading (Lowy, 2003). Assessed expenditure because of multidrug-resistant bacterial infection results in added healthcare charges with loss of outcome (Freire-Moran et al., 2011). The majority of the pharma corporations have the usual routine of antibiotic allocation, which may no longer be effective or missing regulatory sanctions (Levy and Marshall, 2004). According to the findings of the literature research, the cost of AMR is quite expensive and varies greatly by nation (Utt and Wells, 2016). According to a recent World Bank research, antibiotic resistance would increase the poverty rates and has a greater impact on low-income countries than the rest of the world (worldbank.org, 2019). According to studies, global GDP could fall by 1% year by 2050, with developing countries losing 5–7% of their GDP (Utt and Wells, 2016). This proportion equates to between 100 and 210 trillion US dollars (worldbank.org, 2019). By 2050, multidrug-resistant tuberculosis alone might cost the globe $16.7 trillion (tballiance, 2019). The World Bank research shows that global exports are increasing. The scientific report proved that more antibiotic practice may influence the increased frequency of resistant bacteria; however, the limited use of antibiotics still exhibited lower resistance rates. When antibiotics are administered too often or at random, it enhances selective pressure for bacteria to develop resistance (Laxminarayan and Brown, 2001).

Even though the excess use of antibiotics is strictly restricted all over the world, the over-prescription of antibiotics remains the same. Van Boeckel et al. (2015) reported that there will be around 67% rise in antibiotic consumption by 2030, which would nearly double in quickly developing and densely populated countries like Brazil, Russia, India, China, and South Africa (Van Boeckel et al., 2015). In modern medicine, antibiotic treatment is one of the important tactics to combat bacterial infections. The “golden era” of antibiotics extended from the 1930s to the 1960s, which gave rise to several antibiotics (Nathan and Cars, 2014). That era ended as scientists were unable to sustain the pace of antibiotic discovery in the aspect of evolving resistant bacterial pathogens. Constant failure in the discovery of new antibiotics and unlimited use of antibiotics are the influencing factors responsible for the advent of antibiotic resistance (Nathan, 2004). Hence, drug-resistant pathogens are considered the major alarm to healthcare sectors globally.

### Automatous Insight on Phytochemicals to Overcome Drug Resistance

Due to the increasing efficiency of the development and spread of antibiotic-resistant strains, it is very imperative to determine a novel alternative and effective treatment measures to combat drug-resistant pathogens. Consequently, bioactive phytochemicals have been developed as an alternative to conventional antibiotics in combating such antibiotic-resistant pathogen-mediated infections. Many phytochemicals have demonstrated their potential as antimicrobial agents or antibiotic-reverting agents of prevailing antibiotics (Khare et al., 2021). These phytochemicals have proven to be suitable alternatives to address the development of antibiotic resistance associated with conventional antibiotics.

Plants are a rich source of phytochemicals with a great concern for novel drug discovery. In the present era, modern society relies on herbal medicine and ayurvedic medicine to overcome various diseases like impetigo contagiosa (Sharquie et al., 2000), *chronic gastritis* (Gaby, 2001), *tuberculosis* (Mativandlela et al., 2008), pediatric seizures (Akhondian et al., 2007), and urinary tract infections (Jepson et al., 2012). Fundamentally, phytochemicals are the chemical compounds that are synthesized in plant cells themselves to protect them from predators and pathogens. However, only a few of those plants have been explored and investigated (Gurnani et al., 2014). Crude bioactive compounds are extracted or isolated from plants or plant parts to test against various diseases and disorders due to the continuous evolution of resistant microorganisms, which is the prime risk factor to society in the present state of affairs.

Subsequently, therapeutic possibilities for the treatment of various microbial infections have become inadequate, leading to frequent infection and failure to cure or reduce the infection that increases morbidity and mortality, which was evident during the COVID-19 pandemic. Hence, it is needed to develop a novel alternative or complementary antimicrobial drug which is safer and non-toxic to health (Chitsazian-Yazdi et al., 2015). Herbal medicinal plants are a rich source of bioactive phytocompounds, which have potential against various diseases (Shakeri et al., 2018). Many of these plants or phytochemical compounds are proven to be applied in therapeutics. The satisfying medicinal properties of herbal medicinal plants are also active due to the accompanying phytocompounds, such as phenols, terpenoids, alkaloids, carotenoids, flavonoids, isothiocyanates, indoles, monoterpenes, *etc*. (Molyneux et al., 2007).

**Figure 2:**
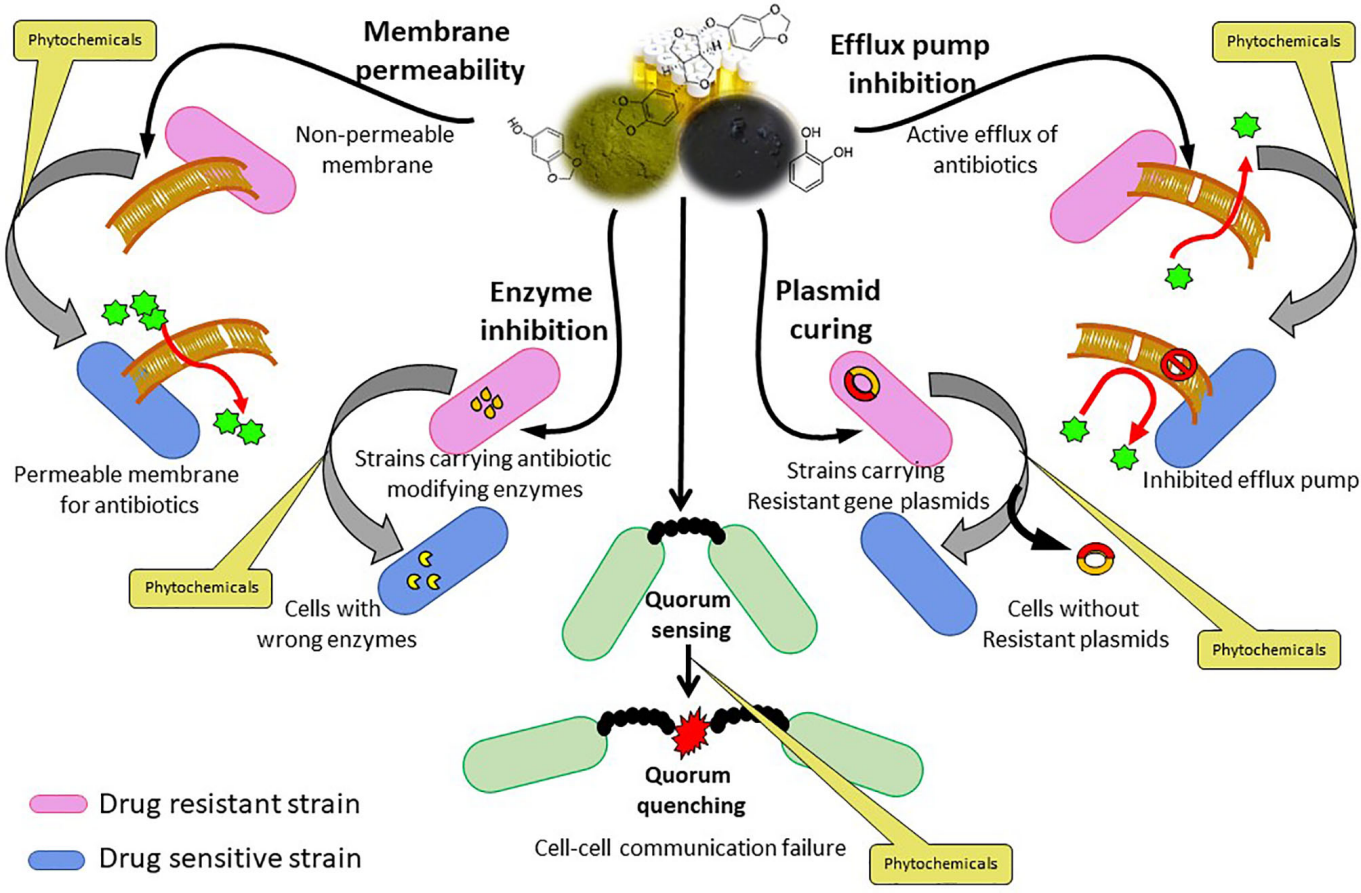
Phytochemicals conferring various bacterial resistance factors.

They have been shown to impede the major resistance-developing factors like efflux pumps, replication machinery, cell microstructure, membrane permeability and integrity, and other virulence mechanisms, including QS and biofilm development, which are essential for the victuals and resistance of pathogenic bacteria (**Figure 2**). Many of the phytochemicals have been ascertained to be effective against drug-resistant strains. Hence, by reviewing the mode of action, these phytochemical agents could pave the way towards the development of novel drugs. Besides bactericidal activity, several plant-derived compounds have also been discovered recently for their potential as adjuvants with antibiotics for re-sensitizing or reverting antibiotic resistance ability. These phytochemicals interfere with the structural membrane by increasing the cell permeability and cellular leakage, through a modification in the bacterial cell wall and cell membrane, resulting in the loss of ATP, attenuation of protein synthesis, destruction of intracytoplasmic, alteration in pH, fragmentation of DNA damage, inhibition of bacterial gene expression, ion binding, inhibition of DNA gyrase, free radical formation drug efflux pumps, mobile genetic elements, QS, and biofilm development (Bazzaz et al., 2018; Yu et al., 2020a).

### Cell Membrane Inhibitors

It is a known fact that bacterial cell membranes act as a protective barrier against antimicrobial agents. Cell membrane permeability regulates the movement of antibiotics into the bacterial cell. It is believed that the mechanism of alteration in the fatty acid and membrane proteins, to monitor the cellular influx of the antibiotics, is reforming their membrane permeability (Yu et al., 2020b). Nevertheless, hydrophobic phytochemicals interact with membrane lipids in such a way to interrupt the cellular structure, eventually leading to higher membrane permeability. This makes bacterial cells unable to monitor the leakage of cellular molecules from the bacterial cells. Several research findings have confirmed the strong abilities of phytochemicals in targeting cell membrane permeability. The altered membrane permeability is possibly attributed to apparent damages to the cellular integrity and functions (Scazzocchio et al., 2017).

### Cell Wall Synthesis Inhibitors

Cells are made up of peptidoglycan, which consists of repeating N‐acetylmuramic acid and N‐acetylglucosamine residues linked together by short chains of amino acids. The amino acid residues are the key components to provide strength and protection to bacteria. The synthesis of bacterial cell walls has been found to be inhibited by several phytochemicals (Upadhyay et al., 2014). The interaction of such phytochemicals with membrane proteins attached to bacterial cell walls eventually leads to an interruption in membrane penetrability. The effective antibacterial potential of phytochemicals belonging to flavonoids in counteracting infectious pathogens is attributed to their ability to complex with bacterial cell walls (AlSheikh et al., 2020).

### Drug Efflux Pump Inhibitor

Bacterial efflux pumps, which diminish the concentration of the administered antibiotics by transporting the antibiotic molecules out of the cell, have evolved as important transporters in drug-resistant strains. As reviewed by Shriram et al. (2008), the bacterial efflux pumps are characterized in two super-families, namely, ATP-binding cassette multidrug transporters and secondary transporters using proton motive force based on their energy source. Further classifications are made based on the secondary transporters, which are further sub-classified into four families based on the substrate specificities; these include major facilitator superfamily, resistance nodulation cell division, multidrug and toxic compound extrusion, and small-MDR family (Putman et al., 2000; Sun et al., 2014). The presence of the efflux pumps in bacterial membranes enables the successful exclusion of the antibiotics out of the cell and thus prevents the active interaction of bacterial targets with antibiotics, leading to the development of resistance. Some phytochemicals are reported as efflux pump inhibitors (EPI) and thus revert antibiotic resistance. The antimicrobial activity of some phytochemicals against bacterial pathogens is conferred by the disruption of bacterial FtsZ Z-ring formation and the subsequent inhibition of bacterial cytokinesis (Kelley et al., 2012).

### Mobile Genetic Elements

Plasmids are mobile genetic elements and are well recognized for transferring resistance genes through horizontal gene transfer among bacterial pathogens. Hence, the elimination of R-plasmid would reduce the transfer of resistance genes among bacteria. The antibacterial as well as resistance reversal potentials of phytochemicals, like essential oils, are attributed to their capability to obliterate R-plasmids. Several phytochemicals with plasmid curing ability have shown strong antibacterial activities when combined with antibiotics like amoxicillin, polymycin, and lincomycin (Si et al., 2008). Hence, the synergistic activity of phytochemicals with conventional antibiotics might possibly reduce the chance of developing drug resistance (Skalicka-Woźniak et al., 2018).

### Enzyme Inhibitors

The antimicrobial potential of several phytochemicals has interconnection with nucleic acid synthesis by blocking the DNA gyrase enzyme which plays a vital role in the replication of DNA molecules (Wu et al., 2013). In some instances, phytochemicals, including flavonoids, are interrupted with helicase (DnaB and RecBCD) activity and hence prevent the DNA replication process (Xu, 2001).

### Targeting Biofilm Formation and Quorum Sensing

Biofilms are the structural community of microbial populations enclosed in an exopolysaccharide matrix (Davey and O’toole, 2000), and their development is regulated by a QS mechanism, in which bacteria can communicate with each other through self-synthesized chemical signals. These signal molecules will be released into the surrounding environment. At threshold concentration, the signal molecule will bind with the appropriate receptor to form a signal receptor complex. Binding of the signal receptor complex with the promoter will, in turn, trigger the expression of virulence factors, such as secretion of virulence enzymes, antibiotic pigment production, extracellular polymeric substance production, and biofilm formation. The mechanisms of biofilm development and QS are reported to be highly effective approaches evolved by the bacteria for conferring drug resistance, its persistence, and spread. Therefore, targeting bacterial biofilms and quorum sensing are emerging as effective approaches for combatting drug resistance. Nevertheless, eliminating or impeding biofilm is challenging. However, several phytochemicals have been reported to exhibit antibiofilm and anti-QS activity. These compounds are considered as novel alternatives to antibiotics towards the prevention of biofilm formation by infectious pathogens. The attenuation of the transcription of genes critical for biofilm formation is attributed to the QS inhibitory activity of phytochemicals (Packiavathy et al., 2014).

### Attenuating Bacterial Virulence

Capsular polysaccharides, produced by some bacteria, are considered as important factors and play a crucial role in the development of virulence (Taylor and Roberts, 2005) as well as to protect the bacteria from phagocytosis (Hyams et al., 2010). Capsular polysaccharides also aid in the adhesion and formation of biofilm. Additionally, capsular polysaccharides aid to enhance the survival rate of pathogens inside the host. Several bacteria displayed a reduced amount of capsular polysaccharide production upon exposure to plant-derived phytochemicals (Derakhshan et al., 2008). They are found effective in reducing the synthesis of capsule secretion by regulating the expression of bacterial regulators of capsule synthesis. Like quorum sensing, adhesion and capsular polysaccharides play a dynamic role in bacterial communication and growth inside the host; it becomes imperative to exploit them for therapeutics for overcoming the burden of increasing antibiotic resistance among microbes.

### Exploring Phytochemicals for Combating Antibiotic Resistance Among Pathogenic Bacteria

Antibiotics comprise a crew of chemotherapeutic agents, either to kill (bactericidal) or to arrest (bacteriostatic) the bacteria to control microbial infections for, *e*.*g*., β-lactam antibiotics, tetracyclines, macrolide antibiotics, aminoglycosides, oxazolidinones, quinolones, lincosamides, cyclic peptides, and sulfa drugs (Gilbert and McBain, 2003). Conversely, the persistent usage of antibiotics is piloted to endure the selective pressures of their environment by the bacteria, resulting in the emergence of multi-drug resistance (Furuya and Lowy, 2006). Antibiotic-resistant infections are becoming a serious issue all over the world. A high proportion of nosocomial infections are instigated by MDR Gram-negative bacteria or by MRSA (Luyt et al., 2014). Similarly, vancomycin-resistant enterococci and an increasing number of bacterial pathogens are developing resistance to several conventional antibiotics (Golkar et al., 2014).

In 2013, the Center for Disease Control and Prevention stated the era as the “post-antibiotic era”, and the WHO warned that the emergence of antibiotic resistance is becoming a serious issue for the human race. Though the pharmaceutical industry developed diverse antibiotics to address resistance issues, the curing proportion of patients was comparatively less, making bacterial infections worse (Spellberg and Gilbert, 2014). As an alternative treatment of the bacterial resistance to antibiotics, plant-based antimicrobial agents displayed an effective role in combatting pathogenic bacteria without emerging resistance to these plant-derived phytochemicals, possibly by exploiting diverse mechanisms of action, which could prevent bacterial adaptation as reported (Essawi and Srour, 2000). The remarkable antimicrobial activity, nontoxic nature, and affordability of the discernible phytochemicals are the basis for their extensive usage as potential antimicrobial agents as well as antiseptics in clinical and industrial settings (Livermore, 2003). In the recent past, they have been employed as a source for the discovery of novel antibiotics in the pharmaceutical sector. It is noteworthy that natural products, in particular, plant extracts in the form of either pure compounds or crude extracts, offer boundless prospects towards the development of novel drug discoveries due to their unrivaled accessibility and chemical diversity.

**Figure 3:**
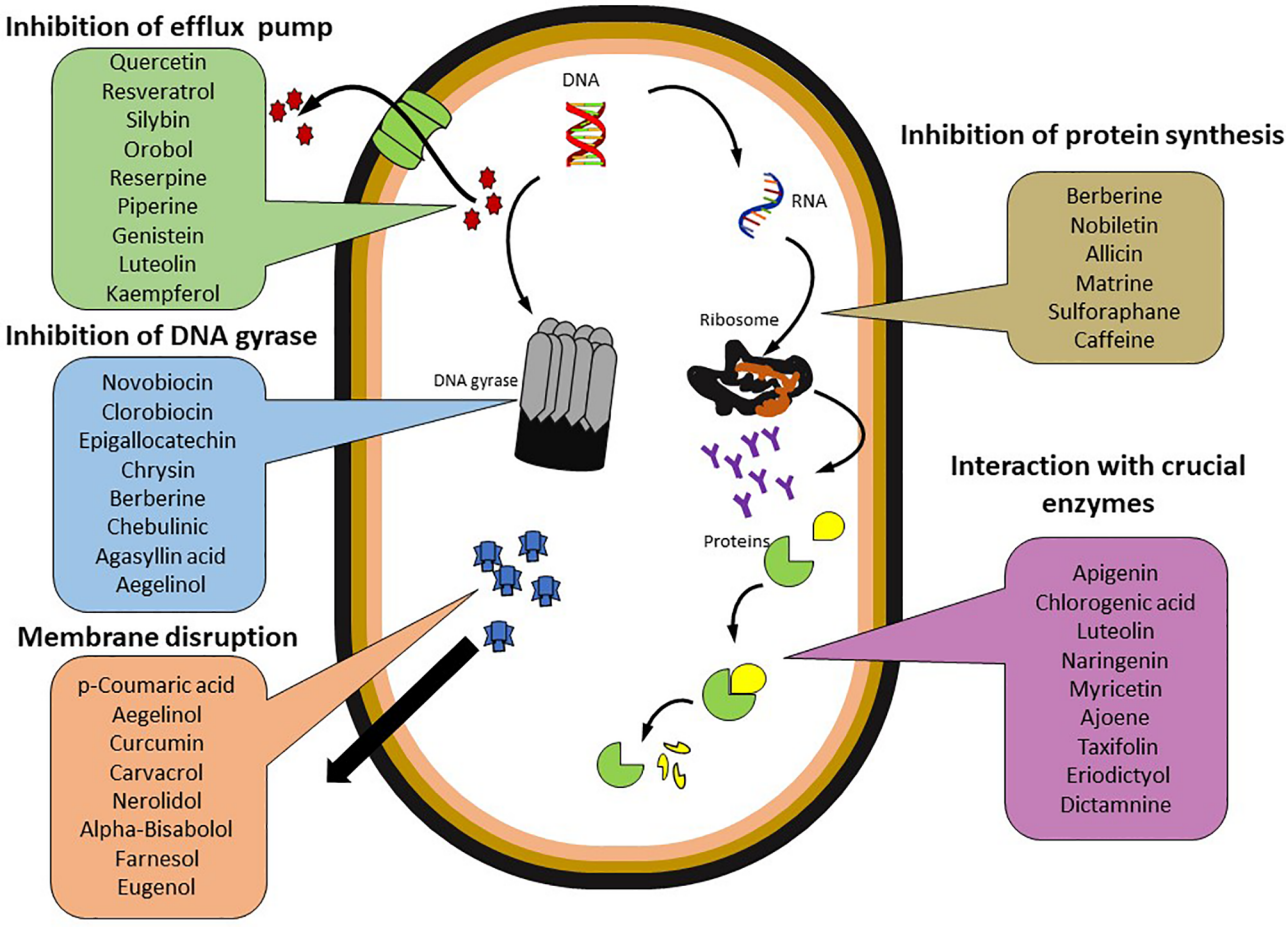
Promising phytochemicals against Multi Drug Resistant bacteria.

The evolution of MDR among bacterial pathogens has directed reconnoitering the perspective of phytochemicals sourced from plants as an alternative therapeutic approach to fight infectious diseases. Among the alternative and potential strategies against MDR pathogens, plant sources possibly play a vital role in offering a vast range of chemicals as secondary metabolites with potent action to combat bacterial infections (Anand et al., 2019; Anand et al., 2020). Such phytochemicals comprise various members of alkaloids, coumarins, flavonoids, quinones, *etc*. (Mbaveng et al., 2015; Anand et al., 2019; Anand et al., 2020; Mohammed et al., 2021). Owing to the potent applications of phytochemicals as antimicrobials, herbal medicines, food enhancements, and cosmetics, they have gained the attention of researchers; hence, several phytochemicals have been endorsed for their effective antimicrobial activities against various pathogenic bacteria, including MDR strains (Shriram et al., 2018; Anand et al., 2019; Yu et al., 2020a; Mohammed et al., 2021). Among the reported phytochemicals, the Food and Drug Administration approved a few of them based on clinical assessments. The effective role of various phytochemicals against multi-drug resistant pathogens has been reviewed (**Figure 3** and **Table 1**).

**Table 1:** Plant based antimicrobial compounds and their mechanism of action.

Class of compound	Phytochemical	Target pathogen	Mechanism of Action	Ref
Alkaloids	Dictamnine	*Saccharomyces cerevisiae*	Inhibiting type II topoisomerase	Heeb et al., 2011; Guo et al., 2008
	Sanguinarine	*Carbapenem-resistant Serratia marcescens*	Inhibiting replication and transcription	Awasthi et al., 2011; Fu et al., 2021
	Chelerythrine	*MRSA* and *Escherichia coli*	Damaging the bacterial cells	He et al., 2018; Wang et al., 2021
	Matrine	*E. coli and Bacillus subtilis*	Inhibiting the synthesis of proteins	Xiu et al., 2017,
	Caffeine	*P. aeruginosa*	Interaction with the quorum sensing proteins and inhibiting biofilm formation	Chakraborty et al., 2019; Anjani et al., 2020
	8-epidiosbulbin E-acetate	*E. coli, E. faecalis, P. aeruginosa* and *S. sonnei*	Plasmid curing	Shriram et al., 2008
	Reserpine	*Staphylococcus sp., Streptococcus sp. and Micrococcus sp.*	Efflux pump inhibitor	Sridevi et al., 2017
	Piperine	*M. resistant, S. aureus* (MRSA) *and S. aureus*	Efflux pump inhibitor	Khameneh et al., 2015
	Berberine	*E. coli* and *C. albicans*	Cell division inhibitor, Protein and DNA synthesis inhibitor	Boberek et al., 2010; Zoric et al., 2017
	Chanoclavine	*E. coli*	Efflux pump inhibitor	Dwivedi et al., 2019
	Solasodine	*C. albicans*	Destruction of bacterial membrane	Chang et al., 2017
	Conessine	*P. aeruginosa* and *M. luteus*	Efflux pump inhibitor	Siriyong et al., 2017
	Tomatidine	*Listeria, Bacillus* and *Staphylococcus spp*	ATP synthase inhibitor	Boulet et al., 2018; Guay et al., 2018
	Lysergol	*E. coli*	Efflux pump inhibitor	Maurya et al., 2013
Organosulfur compounds	Diallyl Sulfides	*C. albicans*	Inhibiting glutathione (GSH) S-transferase (GST) activity. Interaction with the quorum sensing proteins and inhibiting biofilm formation	Velliyagounder et al., 2012; Li et al., 2019
	Allicin	*S. epidermidis, P. aeruginosa* and *S. agalactiae*	Sulfhydryl-dependent enzyme inhibitor, DNA and protein synthesis inhibitor	Reiter et al., 2017
	Ajoene	*C. jejuni, Streptococcus spp, Staphylococcus spp* and *E. coli*	Sulfhydryl-dependent enzyme inhibitor	Rehman and Mairaj, 2013
	Sulforaphane	*E. coli*	Destruction of bacterial membrane, ATP synthase inhibitor, DNA and protein synthesis inhibitor	Wu et al., 2012
Phenolic compounds	Sophoraflavanone G	MRSA	Interacting with peptidoglycan and inhibiting cell wall biosynthesis	Mun et al., 2014
	Acetosyringone	*S. cerevisiae*	Depolarization of the bacterial cell membrane	Saravanakumar et al., 2016; Szatmári et al., 2021
	Chlorogenic acid	*Providencia alcalifaciens, Moraxella catarrhalis, S. aureus*, and *E. coli*	Interacting with some crucial enzymes	Neetu et al., 2020
	Galangin	*S. aureus*	Damaging of the cytoplasmic membrane and inhibition of β-lactamase	Ouyang et al., 2017
	Chrysin	*H. pylori*	Cell membrane disruption, DNA gyrase inhibition	Wu et al., 2013; Lee et al., 2017
	Tannic acid	*S. aureus*	Ion binding	Diniz-Silva et al., 2016
	(+)-Catechin	MRSA	Inhibition of bacterial gene expression	Sinsinwar and Vadivel (2020)
	Resveratrol	*M. smegmatis* and *C. jejuni*	Efflux pump inhibitor	Lechner et al., 2008; Klancnik et al., 2017
	Baicalein	*M. smegmatis*, MRSA and *C. albicans*	Efflux pump inhibitor	Lechner et al., 2008; Chan et al., 2011
	Biochanin A	*M. smegmatis*, MRSA and *Chlamydia spp.*	Efflux pump inhibitor	Lechner et al., 2008; Zou et al., 2014
	Formononetin	*M. smegmatis*	Efflux pump inhibitor	Lechner et al., 2008
	Luteolin	*Mycobacteria spp.* and *M. smegmatis*	Efflux pump inhibitor	Lechner et al., 2008; Rodrigues et al., 2011
	Kaempferol	MRSA and *C. albicans*,	Efflux pump inhibitor	Randhawa et al., 2016; Shao et al., 2016
	Kaempferol rhamnoside	*S. aureus*	Efflux pump inhibitor	Holler et al., 2012a
	Myricetin	*M. smegmatis*	Efflux pump inhibitor	Lechner et al., 2008
	Rhamentin	*S. aureus*	Efflux pump inhibitor	Brown et al., 2015
	Quercetin	*S. aureus*	Efflux pump inhibitor	Brown et al., 2015
			
	Chrysosplenol-D	*S. aureus*	Efflux pump inhibitor	Stermitz et al., 2003
	Chrysoplentin	*S. aureus*	Efflux pump inhibitor	Stermitz et al., 2003
	Silybin	*S. aureus*	Efflux pump inhibitor	Stermitz et al., 2001
	Biochanin A	*S. aureus*	Efflux pump inhibitor	Morel et al., 2003
	Genistein	*S. aureus*	Efflux pump inhibitor	Morel et al., 2003
	Orobol	*S. aureus*	Efflux pump inhibitor	Morel et al., 2003
	4′,6′-Dihydroxy-3′,5′-dimethyl-2′- methoxychalcone	*S. aureus*	Efflux pump inhibitor	Belofsky et al., 2004
	4-phenoxy-4′-dimethylamino ethoxychalcone	*S. aureus*	Efflux pump inhibitor	Holler et al., 2012b
	4-dimethylamino-4′-dimethylamino ethoxychalcone	*S. aureus*	Efflux pump inhibitor	Holler et al., 2012b
	Bergamottin epoxide	MRSA	Efflux pump inhibitor	Abulrob et al., 2004
	5,7-dihydroxy-6-(2-methylbutanoyl)- 8-(3-methylbut-2-enyl)-4-phenyl-2H- chromen-2-one	MRSA	Efflux pump inhibitor	Campos et al., 2009
	5,7-dihydroxy-8-(2-methylbutanoyl)- 6-(3-methylbut-2-enyl)-4-phenyl-2H- chromen-2-one	MRSA	Efflux pump inhibitor	Campos et al., 2009
	Epigallocatechin gallate	*S. aureus*	DNA gyrase, Inhibiting the B subunit of DNA gyrase, penicillinase, and β-lactamase	Gradisar et al., 2007
	Chebulinic acid	*M. tuberculosis*	DNA gyrase	Patel et al., 2015
	3-p-Trans-coumaroyl-2- hydroxyquinic acid	*S. aureus*	Damage to the cytoplasmic membrane	Wu et al., 2016
	p-Coumaric acid	*O. oeni* and *L. hilgardii*	Damage to the cytoplasmic membrane	Campos et al., 2009
	Apigenin	*H. pylori* and *E. coli*	d-Alanine:d-alanine ligase	Wu et al., 2008
	Sophoraflavanone B	MRSA	Direct interaction with peptidoglycan	Mun et al., 2014
	Naringenin	*E. faecalis*	Beta-Ketoacyl acyl carrier protein synthase (KAS) III	Jeong et al., 2009
	Eriodictyol	*E. faecalis*	Beta-Ketoacyl acyl carrier protein synthase (KAS) III	Jeong et al., 2009
	Taxifolin	*E. faecalis*	Beta-Ketoacyl acyl carrier protein synthase (KAS) III	Jeong et al., 2009
	Sakuranetin	*H. pylori*	FabZ	Zhang et al., 2008
	3,6-Dihydroxyflavone	*E. coli*	Beta-Ketoacyl acyl carrier protein synthase (KAS) III and I	Farhadi et al., 2019
	Curcumin	*S. aureus*	Sortase A	Park et al., 2005
		*S. aureus* and *E. coli*	leaky membrane	Tyagi et al., 2015
	Morin	*S. aureus*	Sortase A and B	Kang et al., 2006
	4′,7,8-trihydroxyl-2-isoflavene	*H. pylori*	urease inhibitor	Xiao et al., 2013
Coumarins	Daphnetin	*P. fluorescens* and *Shewanella putrefaciens*	Cell membrane Disruption, Type III secretion inactivation	Yang et al., 2016; Yang et al., 2018
	Esculetin	*Ralstonia pseudosolanacearum*	Cell membrane Disruption, Type III secretion inactivation	Holler et al., 2012a; Yang et al., 2018
	Umbelliferone	*R. pseudosolanacearum*	Cell membrane Disruption, Type III secretion inactivation	Holler et al., 2012a; Yang et al., 2018
	Aegelinol	*S. enterica serovar Typhi, E. aerogenes, E. cloacae* and *S. aureus*	DNA gyrase inhibitor	Basile et al., 2009
	Agasyllin	*H. pylori*	DNA gyrase inhibitor	Basile et al., 2009
	4′-senecioiloxyosthol	*S. enterica serovar Typhi, E. aerogenes, E. cloacae and S. aureus*	DNA gyrase inhibitor	Tan et al., 2017
	Osthole	*H. pylori*	DNA gyrase inhibitor	Tan et al., 2017
	Asphodelin A 4′-O-β-D-glucoside	*B. subtilis*	DNA gyrase inhibitor	El-Seedi, 2007
	Asphodelin A	*B. subtilis, S. aureus*, *K. pneumonia* and MSRA	DNA gyrase inhibitor	El-Seedi, 2007
	Clorobiocin	*S. aureus, E. coli, P. aeruginosa, C. albicans* and *B. cinerea*	DNA gyrase inhibitor	Maxwell, 1993
	Novobiocin		DNA gyrase inhibitor	Maxwell, 1993
	Coumermycin A1		DNA gyrase inhibitor	Maxwell, 1993
	Bergamottin epoxide	MSRA	Efflux pump inhibitor	Roy et al., 2013
	6-Geranyl coumarin	*S. aureus*	Efflux pump inhibitor	de Araujo et al., 2016
	Galbanic acid	MDR clinical isolates of *S. aureus*	Efflux pump inhibitor	Bazzaz et al., 2010
Terpenes	α-Pinene	*H. pylori*	Disrupting cell membrane integrity	Konuk and Ergüden, 2020; Jeyakumar et al., 2021
	Limonene	*S. aureus*	Disrupting cell membrane integrity	Konuk and Ergüden, 2020; Jeyakumar et al., 2021
	Linalool	*P. aeruginosa*	Disrupting cell membrane integrity, changing in the nucleoid morphology, and interfering with cellular respiration	Nguyen et al., 2018;
	Sabinene	Multi drug-resistant strains	Disrupting cell membrane integrity and inhibiting DNA synthesis	Matias et al., 2016
	α-Terpineol	*E. coli*	Lossing membrane-bound autolytic enzymes, the cytoplasm leakage and inability to osmoregulate	Carson et al., 2002; Li et al., 2014
	Citronellol	*Trichophyton rubrum*	Deteriorating membrane integrity	Lopez-Romero et al., 2015; Pereira et al., 2015
	α-Bisabolol	*Propionibacterium acnes* and *S. epidermidis*	Disrupting cell membrane integrity	Sieniawska et al., 2015
	Farnesol	*S. aureus*	Cell membrane disturbance	Togashi et al., 2010
	Nerolidol	*S. aureus*	Cell membrane disturbance	Togashi et al., 2010
	Dehydroabietic acid	*S. aureus*	Cell membrane disturbance	
	(4R)-(-)-carvone	*C. jejuni, E. faecium* and *E. coli*	Cell membrane disturbance	De Carvalho and Da Fonseca, 2006
	(4S)-(+)-carvone	*L. monocytogenes*	Cell membrane disturbance	De Carvalho and Da Fonseca, 2006
	Thymol	*C. albicans*	Inhibits H (+)-ATPase in the cytoplasmic membrane, cell membrane disturb a efflux pump	Sharifzadeh et al., 2018
	Carvacrol	*A. niger, A. fumigatus, A. flavus*, *A. ochraceus, A. alternata, B. cinerea, Cladosporium spp.*, *P. citrinum, P. chrysogenum*, *F. oxysporum* and *R. oryzae*,	Cell membrane disturbance, efflux pump inhibition	Abbaszadeh et al., 2014
	Eugenol	*A. niger, A. fumigatus, A. flavus, A. ochraceus, A. alternata, B. cinerea, Cladosporium spp., P. citrinum*, *P. chrysogenum, F. oxysporum* and *Rhizopus oryzae*	Cell membrane disturbance	Abbaszadeh et al., 2014
	Menthol	*A. niger, A. fumigatus, A. flavus, A. ochraceus, A. alternata, B. cinerea, Cladosporium spp., P. citrinum*, *P. chrysogenum, F. oxysporum* and *R. oryzae*	Cell membrane disturbance	Abbaszadeh et al., 2014
	Cinnamaldehyde	*H. pylori*	Cell membrane disturbance	Ali et al., 2005
	Ursolic acid	*E. coli*	Cell membrane disturbance	Broniatowski et al., 2015
	α-Amyrin	*E. coli*	Cell membrane disturbance	Broniatowski et al., 2015

### Molecular and Biochemical Evidence of Phytochemicals to Treat Bacterial Pathogens

Attenuation of bacterial virulance is considered as a key role of phytochemicals to combat bacterial resistance potential. Interestingly, the chemical structure and the properties of natural phytocompounds reveal their antimicrobial potential by these mechanisms (Khameneh, et al., 2015). Hence, isolation and profiling of the bioactive-rich compounds, such as alkaloids, phenols, flavonoids, terpenoids, *etc*., owing to antimicrobial activity, is an essential part for the development of novel and natural antimicrobial drugs, and they have specific clinical importance due to their bioactivity which does not lead to resistance. Generally, these bioactive compounds are broadly classified as polyphenolics, alkaloids, tannins, glycosides, and steroids. Among these, polyphenols exhibit antimicrobial activity against a wide range of microorganisms. Particularly, polyphenolic compounds, such as flavanol and phenolic acids, were proven to have the greatest activity due to various scientific reasons, including attenuating the virulence factor of bacteria, including enzymes and toxins, dropping the extracellular polysaccharide activity, and performing as extracellular polysaccharide inhibitors. Much scientific research had evidently proved that an increase in the concentration of compounds stimulated the inhibition potential of pathogens (Bazzaz et al., 2018).

### Alkaloids

Alkaloids are a cluster of heterocyclic nitrogenous compounds possessing wide-ranging antimicrobial potential. Alkaloids were proven to be an active antimicrobial agent due to the presence of heterocyclic compounds with highly flexible chemical structures. Alkaloids such as quinolone, dictamnine (Siriwong et al., 2015), and kokusagine, which are isolates of *Teclea afzeli*, showed antibacterial activity by enzymatic alteration, disturbing physiological processes such as restricting DNA synthesis and repair mechanisms (Yan et al., 2021). Many scientific reports suggest that the supreme groups of alkaloids, such as isoquinolines, aporphines, quinolones, and phenanthrenes, show suitable antibacterial activity against a wide range of bacterial pathogens, including *B. cereus*, *S. aureus*, and *K. pneumonia* (Porras et al., 2020), which can inhibit type II topoisomerase enzyme, subsequently hindering DNA replication, and reduce the consumption of O_2_ against bacteria. Plant-derived compounds such as curcumin, tannin, and piperine were proven to possess fantastic antimicrobial potential by directly targeting the DNA or protein. A combination of piperine, which was isolated from *Piper nigrum*, and ciprofloxacin attenuated the development of mutant *S. aureus*. Moreover, the administration of piperin and gentamicin has an inhibitory effect on multidrug-resistant organisms. Diterpenoid alkaloids, commonly isolated from plants, belong to Ranunculaceae and were reported to have antimicrobial properties. The mechanism of action of quaternary alkaloids, such as berberine and harmane, is accomplished by their ability to interpolate with DNA, thus leading to impairment in cell division and subsequent cell death (Boberek et al., 2010). Similarly, berberine has a serious antimicrobial potential against bacteria, fungi, protozoa, and even viruses by aiming at DNA intercalation, affecting RNA polymerase, gyrase, and topoisomerase, and by inhibiting cell division (Yi et al., 2007). The phytochemical compound *Berberis* spp. inhibited the growth of *E. coli* by blocking the synthesis of cell division and protein and DNA synthesis (Boberek et al., 2010). The antimicrobial compound chanoclavine, which was isolated from *Ipomoea muricata*, had shown synergistic activity when co-administered with tetracycline, which seems to inhibit EP, and reported as being effective and ATPase dependent (Dwivedi et al., 2019). Maurya et al. (2013) reported *I. muricata*-derived lysergol against *E. coli* by targeting the efflux pump. Another efflux pump inhibitor, reserpine, extracted from *Rauwolfia serpentina*, showed antimicrobial activity against Gram-positive pathogens *Staphylococcus* spp. and *Streptococcus* spp. Similarly, conessine, an alkaloid compound isolated from *Holarrhena antidysenterica*, displayed a potent inhibitory activity against *P. aeruginosa* by inhibiting the bacterial efflux pump (Siriyong et al., 2017). Sanguinarine, a benzophenanthridine alkaloid originating from the rhizomes of *Sanguinaria canadensis*, exhibited antimicrobial and anti-inflammatory properties. The antibacterial activity exhibited by this molecule is accomplished by the intrusion of bacterial cytokinesis (Kelley et al., 2012). The synergistic effect of this compound with vancomycin, and EDTA was found to be effective against Gram-negative bacteria (Hamoud et al., 2015). In MRSA strains, sanguinarine enables the release of membrane-bound cell wall autolytic enzymes, resulting in cell disruption (Obiang-Obounou et al., 2011).

Plasmid, a self-replicating, circular DNA coding for various gene groups, exhibits antibiotic resistance to bacteria. Some phytochemicals have been reported to target such plasmids (Buckner et al., 2018). 8-Epidiosbulbin-E-acetate, from *Dioscorea bulbifera*, is ascertained to cure the antibiotic-resistant R-plasmids of the clinical isolates of *E. faecalis*, *E. coli*, *Shigella sonnei*, and *P. aeruginosa* with effective curing efficacy (Shriram et al., 2008). Tomatidine, derived from Solanaceous plants, was documented to display antibacterial activity against *Listeria*, *Bacillus*, and *Staphylococcus* spp. The possible mechanism of action of tomatidine is postulated as an ATP synthase inhibitor (Guay et al., 2018).

### Organosulfur Compounds

Allicin, an organosulfur compound from *Allium sativum*, has antibacterial activity against *P. aeruginosa* and *S. epidermidis.* The antibacterial action mechanism of allicin includes DNA synthesis inhibition, protein synthesis inhibition, and sulfhydryl-dependent enzyme inhibition (Reiter et al., 2017). Similarly, the investigation by Rehman and Mairaj (2013) suggested that the antimicrobial action of ajoene, from *A. sativum*, inhibits the sulfhydryl-dependent enzyme inhibitor of *Campylobacter jejuni*. The use of *Diplotaxis harra-*derived sulforaphane as an ATP synthase inhibitor and DNA/protein synthesis inhibitor was examined, and the results revealed that this compound effectively arrests the growth of *E. coli*. Furthermore, this compound has also been proven to destroy the membrane of the target pathogen (Li et al., 2017).

### Phenolic Compounds

Phenolic compounds from plants are considered imperative molecules for drug discovery due to their broad spectral and important medicinal properties. The structure of phenolic compound plants includes an aromatic ring with one or more hydroxyl groups, and these are grouped into flavonoids, phenolic acids, and non-flavonoids (de Souza et al., 2019). They have been recognized as potent chemopreventive and therapeutic agents against diverse pathogenic bacteria and act as natural antimicrobial weapons by enhancing the sensitivity of MDR strains to antibiotics (Miklasińska-Majdanik et al., 2018; Makarewicz et al., 2021). Most notably, by reducing EP activity as the most significant mechanism, phenolic acids play a vital role in attenuating the resistance potential of various pathogens. Compounds such as resveratrol and flavanol are capable of inhibiting the activity of CmeABC Eps of *C. jejuni* or Eps of *M. smegmatis* (Klancnik et al., 2017). Furthermore, ferulic acid derivatives, 4-[E-2-(diethylcarbamoyl) vinyl]-2- methoxyphenyl acetate (E)-methyl 3-{4-[(p-tolylcarbamoyl) methoxy]-3-methoxyphenyl} acrylate, were found to exhibit antibacterial activity against MRSA by inhibiting the efflux pump (Sundaramoorthy et al., 2018). A similar kind of EPI activity was displayed by baicalein (Chan et al., 2011), kaempferol (Randhawa et al., 2016), and resveratrol (Klancnik et al., 2017) against MRSA and *C. jejuni*, respectively. The phenolic compound was also acknowledged as a beta-ketoacyl acyl carrier protein synthase inhibitor. As an example, taxifolin, from *Allium cepa*, showed an effective antibacterial activity against *Enterococcus faecalis* (Jeong et al., 2009).

Polyphenols (tannins) (Gradisar et al., 2007), chebulinic acid (Patel et al., 2015), and anthraquinones (Duan et al., 2014) are natural phenolic compounds that exhibit inhibition against DNA gyrase. Wu et al. (2016) revealed that a unique phenolic compound, 3-p-trans-coumaroyl-2-hydroxyquinic acid, extracted from *Cedrus deodara* showed antibacterial activity against 11 foodborne organisms. The mechanism of action of resistance against *S. aureus* would possibly cause damage to the cytoplasmic membrane and thereby cellular leakage of intracellular organelles due to hyperpolarization with loss of membrane integrity. It was believed that this CHA would be a better antimicrobial agent for the food and beverage industries. In general, compounds such as hydroxycinnamic acids (p-coumaric, caffeic, and ferulic acids) are other phenolic compounds that are capable of affecting membrane integrity. However, similar compounds, like p-coumaric acid, are believed to be the first prior compounds to have a potential activity due to their lipophilic nature (Campos et al., 2009; Wu et al., 2016). The results from Lanzotti et al. (2014) revealed antimicrobial activity accounting for the prevention of sulfhydryl-dependent enzymes, like alcohol dehydrogenase, thioredoxin reductase, and RNA polymerase, which was established by identifying the reduced inhibitory effect of allicin caused by the addition of cysteine and glutathione in the medium, reacting with its disulfide bond and resulting in the prevention of cellular damage. Besides this, allicin was proved to be an inhibitor of DNA and protein synthesis, which would be a possible target of allicin (Lanzotti et al., 2014). Phenolic compounds, such as pyrogallol and catechol, have been examined to show antimicrobial activity against a wide range of Gram-positive and Gram-negative bacteria. Pyrogallol and pyrocatechol were found to be effective against various oral pathogens (Shahzad et al., 2015). Additionally, halogenated catechols have also been investigated for their antimicrobial potential against various MDR strains by impeding the fatty acid synthesis of pathogenic bacteria (Liu et al., 2021). Borges et al. (2013) investigated the antibacterial activity of ferulic acid where it was found to be effective against *P. aeruginosa* and *E. coli* at MICs of 100 μg/ml. Similarly, gallic acid displayed antibacterial properties against *Listeria monocytogenes*, *P. aeruginosa*, and *S. aureus*. The antibacterial activity of ferulic acid and gallic acid is attributed to their ability in disrupting the cell walls of the target pathogens, leading to local damage and subsequent cellular material leakage. Similarly, gymnemic acid inhibited the biofilm development of *Candida albicans* and *Streptococcus bordonii* (Veerapandian and Vediyappan, 2019).

### Flavonoids

Plant flavonoids are phenolic compounds holding a 2-phenyl-benzo-γ-pyrane nucleus and two benzene rings with potent antimicrobial activities. The various groups of flavonoids, such as flavanols, flavanones, isoflavonoids, chalcones, and dihydrochalcones, have been reported to exhibit antimicrobial properties (Górniak et al., 2019). Catechin causes membrane disruption in MRSA, which results in cell membrane damage by leakage of potassium ions. Budzyńska et al. (2011) analyzed the 3-arylideneflavanone-mediated membrane disruption, which leads to the accumulation of bacterial cells, resulting in the alteration of membrane integrity and enabling the increased permeability of pathogenic *S. aureus* and *E. faecalis* isolated from clinical samples. Interference of DNA synthesis activity was reported with flavonoid, chrysin, and kaempferol (Wu et al., 2013) and morin and myricetin (Xu, 2001). Some flavonoids have been reported as sensitizing agents. The combination of pinostrobin-a with antibiotic ciprofloxacin exhibited a synergistic effect to enhance the growth inhibitory potential of antibiotic-resistant strains *P. aeruginosa* and *E. coli* by blocking the EPI activity (Christena et al., 2015). Flavonoids are also recognized as inhibitors of quorum sensing and biofilm formation. Ouyang et al. (2016) demonstrated the QS and biofilm inhibitory activity of quercetin in *P. aeruginosa PAO1.* The QS activity of quercetin is attributed to the attenuated expressions of lasI, lasR, rhlI, and rhlR genes with decreased secretion of virulence factors like elastase, protease, and pyocyanin.

### Terpenes

Terpenes, also called isoprenoids, are the largest single class of compounds present in essential oil and are made up of isoprene molecules (Praveen, 2018). Essential oils (EOs) consist of a combination of various phytochemicals and are highly recognized for their effective antimicrobial activity. Additionally, they have been employed as a traditional medicinal treatment to encounter antibiotic resistance since they are considered safe to consume and essential for host tissues (Yu et al., 2020b). Cox et al. (2001) reported the increased permeability of bacterial membrane upon treatment with EOs derived from *Melaleuca alternifolia*. Farnesol, a phytochemical isolated from essential oils, inhibited the growth of *S. aureus* by disrupting the cell membrane (Togashi et al., 2010). Methyl eugenol, present in the EOs of *Cumium cymium*, inhibited the biofilm formation and associated virulence of Gram-negative bacterial pathogens like *P. aerugiosa*, *E. coli*, *Proteus mirabilis*, and *Serratia marcescens* by attenuating the signal-based QS (Packiavathy et al., 2012). Similarly, the biofilms of uropathogenic bacteria demonstrated altered biofilm patterns in the presence of the quorum quencher molecule, *Curcuma longa*-derived curcumin (Packiavathy et al., 2014). The EOs of cinnamon displayed effective bactericidal activity against *E. coli* and *Staphylococcus* strains by altering the membrane permeability and structural integrity (Zhang et al., 2015). The EOs extracted from *Coriandrum sativum* inactivated the MDR uropathogenic *E. coli* strain by interrupting the cell membrane permeability (Scazzocchio et al., 2017). The striking antimicrobial activity of *Plectranthus amboinicus*-derived EOs against drug-resistant *S. aureus* is attributed to its biofilm inhibitory potential (Vasconcelos et al., 2017). The striking biofilm and QS inhibitory potential in reverting the resistance of *S. aureus* is attributed to the EOs of *Satureja hortensis* (Sharifi et al., 2018). An important compound, cis-cis-p-menthenolide, present in the EOs of *Mentha suaveolens* ssp. *insularis* was found to inhibit the signal-mediated QS system and biofilm formation of *Chromobacterium violaceium*. This compound exhibited a structural similarity to the natural signal molecule and hence acts as a competitive inhibitor, which could lead towards the blocking of gene expression and succeeding biofilm formation (Poli et al., 2018). EOs from *Cinnamomum verum*, *Thymus vulgaris*, and *Eugenia caryophyllata* were found to inhibit the growth of several MDR clinical isolates through the inhibition of biofilm and QS activities (Al1ibi et al., 2020). Very recently, a study by Önem (2022) displayed the QS-mediated biofilm inhibitory potential of *Cymbopogon martini* EOs and proved that the activity of these EOs is attributed to the phytochemical molecule geraniol.

As these phytochemicals have proved to inhibit the major resistance-creating factors such as efflux pumps, replication machineries, cell permeability, biofilm formation, and QS inhibition, they are considered crucial promising alternatives to overcome the decreasing activity of conventional antibiotics. The combinatorial application of these phytochemicals has proved to be highly effective against antibiotic-resistant strains. Hence, there is a pressing need for advanced research, scientific endorsement, and application of these phytochemicals to combat MDR pathogens.

### Preclinical and Clinical Studies on the Antibacterial Effect of Phytochemicals

The transformation of *in vitro* studies to *in vivo* investigations and, finally, to human clinical trials is a great task in the improvement of novel phytocompounds. Various phytochemical medicinal plants exhibited antimicrobial activity, which can act as an alternative treatment to conventional medicine. However, it is expensive and time-consuming to bring a new novel drug/antibiotic to the market. Hence, the isolation of drugs from natural sources had extended its importance in the identification of chemical compounds with resistance properties (Mandal et al., 2014). The preclinical and clinical analysis guidelines for phytochemical compounds are required to safeguard the consistency in drug formulation, their efficacy, and their safety. Compounds isolated from herbal medicines, which were preclinically tested, against various infectious diseases and then licensed by completing the preclinical studies. It may be either one compound or two or more bioactive constituents being co-administered. Despite there being a vast number of bioactive compounds identified in recent centuries, only a few of them are examined *via* clinical studies. Moreover, most of the phytocompounds, when used as monotherapy, require a higher concentration in comparison with antibiotics. To address these problems, researchers focused on the combination of increased phytochemicals with less synthetic antibiotics to inhibit the resistance activity against various microbes (Touani et al., 2014; Santiago et al., 2015). To overcome the time consumption of these active phytocompounds as a drug on the market as a part of preclinical studies, *in silico* approach with natural phytocompounds were chosen on the basis of its bioactive constituents. Besides to interpret the characteristics of molecular structures such as the interactions of protein–ligand binding, an analysis of the quantitative structure activity by QSAR helps predict the compound with a specific target (Ahamad et al., 2017). Similarly, studies on pharmacophore models that simulate the 3D arrangements of particles with various physicochemical features are tangled in the interaction between ligand and target. A very common *in silico* approach is molecular docking, which proposes the structure–activity relationship on phytocompounds for revealing its mechanism of action and understanding the positioning of a ligand inside a protein-binding pocket (Fakhrudin et al., 2010; Zhang et al., 2011). Earlier studies revealed that more than 16,000 antimicrobial studies were registered in ClinicalTrials.gov from the year 2000. In approximately only 1 of all 10 registered scientific investigation studies were antimicrobial mediators assessed and investigated. The most common was interventional trials of drugs and biologicals, in which around 75% were randomized and about 26% were recruited for children along with adults. Diagonally between all completed interventional drug trials, only 12% had been rationalized through the investigation results (Stockmann et al., 2013). In agreement with the earlier reports, there are also some pharmacokinetic/pharmacodynamics evaluation studies which were registered in ClinicalTrials.gov since this is an essential and important component in safety and efficacy studies (Ross et al., 2012).

A standardized herbal concentration of “Tokoro Combination” and “Rehmannia and Akebia” was formulated as small granules. Both of these medicines were already approved by the ministry of health and welfare. These drugs consist of major compounds like diosgenin, yamogenin, betulin, oleanolic acid, hederagenin, akeboside, β-sitosterol, stigmasterol, inositol, catalpol, and glycyrrhizin. In the investigation of Girón et al. (1988), it was reported that the combination of *Solanum nigrescens* extract with nystatin showed better results in women. Both were provided as intravaginal suppositories in patients with long-established *C. albicans* vaginitis. The plant extract proved to be more effective when compared with nystatin. Similarly, cranberry juice was given for urinary tract infection, which was investigated in a team of elderly women who showed less bacterial infection in their urine than the untreated control groups (Avorn, 1996). A group of diverse ayurvedic formulations was examined against a placebo for their potency against acne vulgaris. Among these, Sunder Vati’s product revealed a significant reduction of lesion count in comparison with the other three formulations. Compounds such as Provir and Virend were clinically investigated against respiratory viral infection and topical antiherpes agents in 1994, and their safety and efficacy were studied in phase II studies. The extract of *Opuntia streptacantha* exhibited *in vitro* antiviral activity and was found to be safe in mice and humans (King and Tempesta, 2007), yet another compound, berberine, was proved to have a good result against various infections. A concentration above 64 µg/ml exhibited better results and was retained in the intestine, reaching an extraordinary benefit for intestinal infectious diseases and diarrhea (Lin et al., 2018). In the same way, there have been reports that *Houttuynia cordata* Thunb. has a medicinal property against various diseases, such as suppuration, sores, pustules, and respiratory infections, in Chinese pharmacopeia. A compound named houttuynin, which was isolated from *H. cordata*, exhibited antibacterial activity. The compounds isolated were used alone or in combination with conventional antibiotics to battle against infectious diseases (Hou et al., 2018; Liu et al., 2021).

## Conclusion

As the emergence of antibiotic resistance among bacterial pathogens is becoming a major problem in treating infectious diseases, the progression of novel alternative treatment methods is therefore evolving rapidly against drug-resistant pathogens all over the world. As an alternative, phytochemicals have been employed to combat such infections instigated from antibiotic-resistant pathogens. So far, several plant-derived bioactive compounds (phytochemicals) have been reported for their bactericidal as well as antibiotic reversal potential. The bioactive potentials of such phytochemicals have been found to impede the important virulence factors associated with resistance development, such as cell permeability, efflux pumps, DNA replication mechanisms, and other processes linked with bacterial virulence, including biofilm formation and quorum sensing. Moreover, the synergistic effects of these phytochemicals with conventional antibiotics were found to be very effective against antibiotic-resistant pathogenic bacteria. Ultimately, several studies have proved the efficacy of phytochemicals as future drugs, the conversion success, and the scanty commercial use. Therefore, extreme progress is needed towards the commercialization of phytochemicals as proven drugs to encounter MDR-associated infections.

## Author Contributions

Conceptualization: DAA. Writing—original draft: TS, IASVP, GSBA, VR, and NRD. Writing—review and editing: TS, IASVP, GSBA, SM, and DAA. Language correction and editing: AC. Supervision: DAA. All authors contributed to the article and approved the submitted version.

## Conflict of Interest

The authors declare that the research was conducted in the absence of any commercial or financial relationships that could be construed as a potential conflict of interest.

## Publisher’s Note

All claims expressed in this article are solely those of the authors and do not necessarily represent those of their affiliated organizations, or those of the publisher, the editors and the reviewers. Any product that may be evaluated in this article, or claim that may be made by its manufacturer, is not guaranteed or endorsed by the publisher.
